# Application of Wine and Olive Oil Production Residues as Substrates for the Cultivation of *Chrysanthemum morifolium* Potted Plants

**DOI:** 10.3390/plants14081166

**Published:** 2025-04-09

**Authors:** Georgios Toumazou, Munoo Prasad, Antonios Chrysargyris

**Affiliations:** Department of Agricultural Sciences, Biotechnology and Food Science, Cyprus University of Technology, Limassol 3603, Cyprus; giorgostoumazou99@gmail.com

**Keywords:** olive-mill wastes, grape-mill wastes, peat alternatives, ornamentals, soilless cultivation

## Abstract

Peat is widely used as a soilless growing medium due to its favorable physicochemical properties. However, its extraction has a significant environmental impact, highlighting the need for sustainable alternatives. Repurposing residues from olive oil (OR) and wine (GR) production offers a potential solution to reduce peat dependency and promote agricultural circularity. This study investigated the effects of incorporating different ratios of OR and GR (0, 5, 10, 20, and 40% *v*/*v*) into peat-based substrates for the cultivation of chrysanthemum (*Chrysanthemum morifolium* cv. Pina Colada). The addition of OR and GR altered the physicochemical properties of the substrate mixtures. All mixtures maintained electrical conductivity below the maximum threshold for container media (≤0.5 mS cm^−1^). While GR increased pH, it remained within suitable ranges at 5–10% incorporation. Adding OR decreased total porosity, while GR addition at ≥20% increased it. OR-amended substrates were associated with reduced plant growth, flower production, chlorophyll fluorescence, and relative chlorophyll content, with these effects intensifying at higher OR levels. These outcomes, combined with increased total phenolics, flavonoids, antioxidant activity, and antioxidant enzyme activities, suggest a high stress response, as indicated by increased malondialdehyde and hydrogen peroxide levels. In contrast, GR at ≤ 20% did not induce oxidative stress or negatively affect growth, physiological, or nutritional indices, making it a viable component of peat-based substrate mixtures. The suboptimal performance of OR highlights the need for improved valorization through composting, optimized application rates, and combination with other substrates or residues to enhance its suitability as a horticultural substrate component.

## 1. Introduction

Peat comprises nearly 80% of the growing media used in horticulture across Europe [1], primarily due to its favorable chemical, physical, and biological properties. These include high air space and drainage, low nutrient content, low pH, and high water-holding capacity [2,3]. However, its extensive use has led to unsustainable extraction, raising concerns about the environmental impact of draining and mining peatlands, which are depleting much faster than they can regenerate. This degradation results in oxidation and the release of carbon dioxide into the atmosphere [4]. Peat extraction within the European Union contributes up to 12.6 Mt CO_2_-eq, with annual global emissions exceeding 2 Gt CO_2_-eq [3,5]. Additionally, the depletion of peatlands reduces groundwater quality and disrupts ecosystems, affecting wild plants and animals [6]. To mitigate these environmental impacts, researchers and industry professionals have been actively exploring alternatives to reduce peat usage, either through complete or partial substitution with sustainable materials such as organic biochar, coir, wood fibers, and green compost [7,8]. This has driven efforts to identify low-cost, sustainable materials, with properties comparable to peat [9,10,11].

A promising approach to minimizing the complications associated with peat use is the valorization of organic residues generated by agricultural crop production as horticultural substrate constituents [12,13,14]. In the Mediterranean basin, agrifood residues are largely derived from olive tree cultivation and vineyards [15,16,17]. These residues occur at various stages in the olive oil production and winemaking chain. In olive cultivation, considerable waste is generated during the manufacturing phase, with mill waste being a principal environmental issue [18]. These wastes include olive-mill wastewater, two- and three-phase olive-mill waste, and olive pomace [19]. These olive-mill residues (ORs) are environmentally relevant, as they contain large amounts of organic matter, such as phenols, and can also carry phytotoxic and antimicrobial substances [20,21]. On the other hand, wine production generates significant amounts of grape-mill residues (GRs), including grape stalks (peduncles and main stems), grape marc (skin, pulp, and seeds), sediments from the fermentation of wine components, and wastewater [22]. Both waste streams represent a significant environmental concern, and repurposing them contributes to reducing resource consumption and waste generation, aligning with the principles of the Circular Bioeconomy policy [14,23].

Research has prioritized assessing and utilizing sustainable and low-cost materials with suitable characteristics and uniform quality [1,24]. However, despite the volume of data on peat alternatives, research is still at a preliminary stage and establishing suitable materials and proportions for peat substitution remains challenging [1,25]. In addition, the viability of organic materials as substrate constituents depends on the cultivated species, as factors such as the electrical conductivity (EC) and toxic element accumulation may limit their efficacy. In agricultural production, a portion of these wastes is typically composted [26]. However, organic wastes require lengthy processes of aging or composting to convert them into mature, stable products [27]. In some cases, raw (uncomposted or partially composted) organic materials have also been proposed for peat substitution. These include the use of fresh rice hulls [28], spent mushroom substrates [29], and spent coffee grounds [30,31]. The use of OR and GR materials has also been evaluated, though to a limited extent [32,33]. However, their unpredictability in terms of quality, composition, and physicochemical properties constrains their use as raw growth media components [32]. Key factors include chemical (EC levels, pH, cation exchange capacity, accumulation of toxic components), physical (bulk density, water-holding capacity), and biological (absence of pathogens) characteristics that may influence the final substrate mixture’s efficacy [7,34]. Thus, the ratio of these components should be adjusted to reduce potential hazards [6].

The present study focuses on using OR and GR as alternative substrates for the soilless cultivation of chrysanthemum (*Chrysanthemum morifolium*). Chrysanthemum is an herbaceous perennial flowering plant grown worldwide, known for its charming flowers and excellent vase life. It is one of the most important ornamental plant species, both economically and aesthetically, second only to roses, and is available as cut or potted flowers [3,35,36]. Given the economic importance of chrysanthemum and the intensive use of peat in its cultivation, exploring more sustainable substrates while ensuring optimal productivity and value is necessary [37]. Various studies have examined the use of organic materials such as rice husks, spent growing media, leaf mold, and fermented peanuts shells, as peat substitutes for the soilless cultivation of chrysanthemum, with varying degrees of success [3,37,38]. However, using OR and GR as alternatives to peat in chrysanthemum cultivation remains largely unexplored. Thus, this study aimed to evaluate the partial substitution of peat with OR and GR in chrysanthemum production and quality, while also assessing their impacts on nutrient status, antioxidant capacity, and plant stress response to determine the optimal growing conditions using these two materials.

## 2. Results

The physicochemical properties of growing media with OR and GR, before chrysanthemum cultivation, are presented in Table 1. The tested raw residues were characterized by diverse physicochemical characteristics, contributing to the properties of the prepared mixtures. OR had a lower pH compared to GR (6.57 and 7.19, respectively), contributing to a pH close to peat (6.44). For the substrate mixtures containing OR, pH remained similar among CON (100% peat), OR 10%, and OR 20% treatments, while OR 5% and OR 40% exhibited higher pH levels than the other treatments. Additionally, the increasing amount of GR progressively increased the pH of the substrate mixtures. The raw OR material had a higher EC (1006.60 μS cm^−1^), and thus, the presence of ≥10% OR increased the EC of the mixtures compared to CON and lower levels of OR (5%). Although lower, the EC of the raw GR material (767.05 μS cm^−1^) caused an increase in the EC of the GR mixtures at ≥20%, compared to CON and lower levels of GR (5, 10%). When OR was added to the mixture, organic matter and organic carbon were comparable to CON, with the exception of OR 5%, which decreased them. However, GR addition decreased the amount of organic carbon and organic matter, compared to CON. Similarly, the C/N ratio was decreased with the addition of OR, and a gradual decrease was observed with increasing GR levels. The OR-based media exhibited increased N content compared to CON, while no significant differences were found with the increasing addition of the material. K was progressively increased with the increasing addition of OR, whereas P, Ca, Mg, and Na showed a corresponding decrease as the amount of OR increased. For the GR-based media, the observed N, P, K, and Na contents were proportionally increased with increasing amounts of the material, while Ca and Mg exhibited a downward trend (Table 1). The OR residues reduced the total pores’ space, and AFP in most cases, while as the proportion of OR increased, BD demonstrated a consistent increase. AWHC remained unaffected. Finally, the GR residues increased TP and BD at ≥20% GR, and AFP at ≥5% GR.

Following the termination of chrysanthemum cultivation, several substrate media properties were affected (Table 2). For instance, an increase in pH was observed with the addition of OR and GR, becoming more prominent with higher amounts of these materials. EC decreased under OR 10% and OR 20%, while the addition of GR was generally associated with increases at GR ≥ 20%. With OR, the N and K contents were gradually increased with the volume of the material rose, whereas Na showed an opposite trend. In addition, Mg and Ca were decreased with the addition of OR at ≥10%. Interestingly, P was increased with OR 5% compared to CON and the other OR levels. In the case of GR, a positive linear relationship was observed between the amount of the material used and the contents of N, P, and K. In contrast, the contents of Ca, Mg, and Na decreased with increasing levels of GR in the final substrates (Table 2).

The addition of OR and GR to peat-based substrate media influenced the growth of chrysanthemum (Table 3). OR decreased plant height, compared to CON, and plant height was further reduced at higher OR levels (i.e., OR 20 and 40%). Similarly, shoot diameter was negatively affected by OR addition, with the lowest value observed at OR 40%, while leaf number was significantly lower using OR, regardless of the percentage. The fresh and dry weights (FW and DW) of plants leaves, shoots, and flowers decreased with OR addition, compared to CON, regardless of the OR percentage. However, DM was positively correlated with increasing OR levels, exceeding that of the CON treatment. Finally, flower production was generally reduced with OR addition compared to CON, with higher OR levels (i.e., OR 20 and 40%) causing further reductions in closed and total flowers. In stark contrast, plants grown with GR exhibited better performance. In fact, with GR levels below 40%, plant height and shoot diameter did not differ significantly from plants grown with CON. The presence of GR 10% increased the number of leaves produced compared to CON and the other GR treatments. The development of lateral shoots, as well as the FW and DW of plants, leaves, and shoots, were all positively correlated with 5–10% GR, compared to CON. However, at higher GR levels (i.e., GR 20 and 40%), the FW and DW of flowers decreased (Table 3). Similarly, at GR 40%, the number of closed and total flowers was reduced compared to the other treatments.

**Figure 1:**
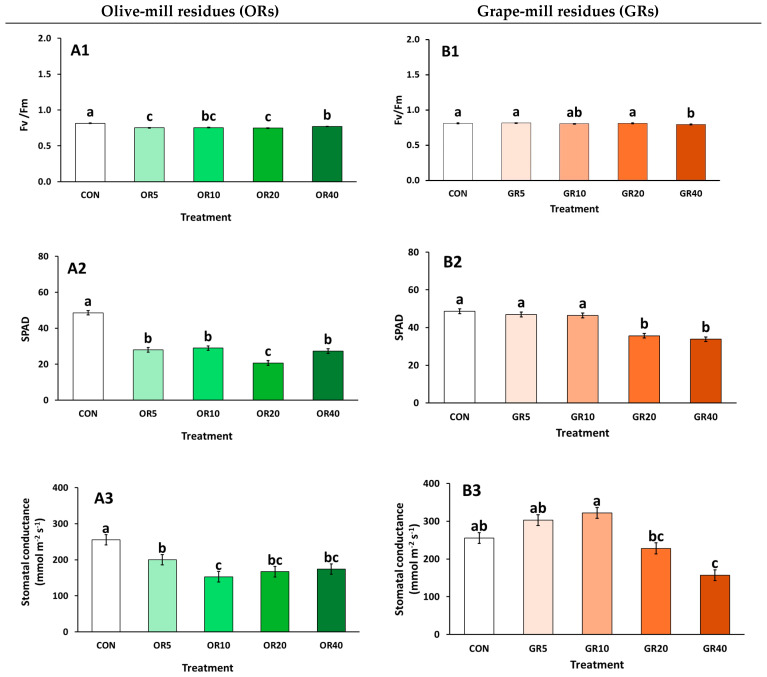
Impact of growing media (peat: CON; olive-mill residues: OR; and grape-mill residues: GR) on leaf chlorophyll fluorescence (Fv/Fm), SPAD, and stomatal conductance (mmol m^−2^ s^−1^) of chrysanthemum (*Chrysanthemum morifolium* cv. Pina Colada) plants (OR: (**A1**–**A3**), GR: (**B1**–**B3**)). Bars indicate the mean ± SE (*n* = 6). Bars with the same letter do not differ significantly at *p ≥* 0.05 according to Duncan’s MRT.

Chlorophyll fluorescence (Fv/Fm) was negatively influenced by the addition of OR, with the lowest values being observed at OR 5% and OR 10% (Figure 1(A1)). Additionally, SPAD was reduced under OR treatments compared to CON, while OR 20% had the lowest SPAD values compared to the rest of the treatments (Figure 1(A2)). Stomatal conductance followed a similar trend, with OR 10% producing plants with the lowest values (Figure 1(A3)). In the case of GR addition, Fv/Fm did not differ significantly with 5–20% GR and CON, while it was reduced with the application of GR 40% (Figure 1(B1)). Similarly, increased levels of GR (i.e., GR 20 and 40%) significantly decreased SPAD and stomatal conductance (Figure 1(B2,B3)).

**Figure 2:**
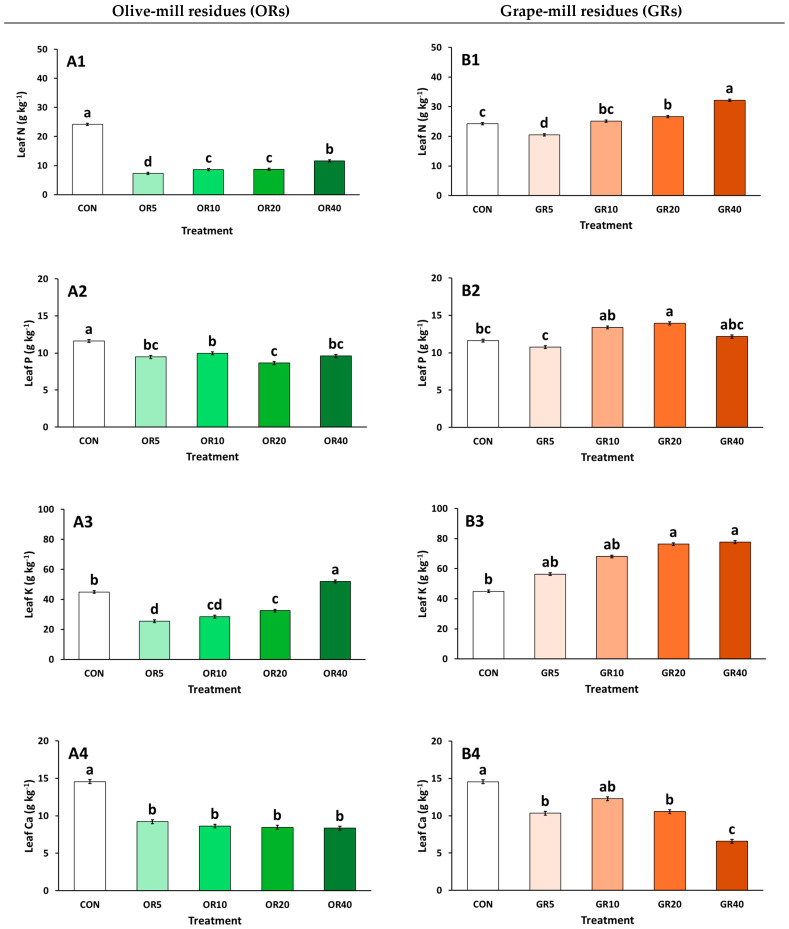

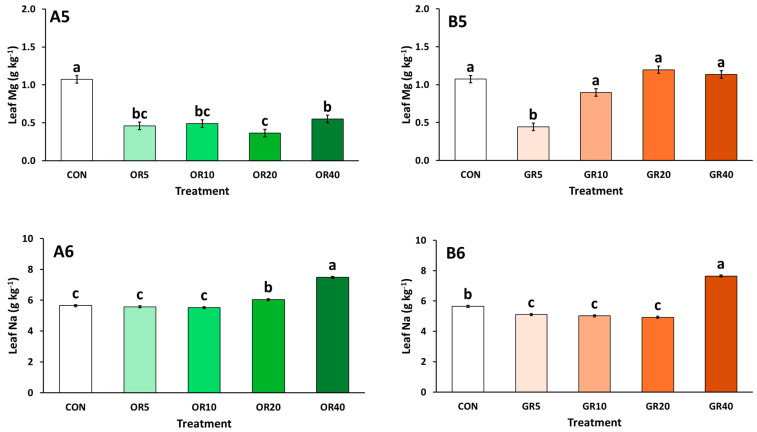
Impact of growing media (peat: CON; olive-mill residues: OR; and grape-mill residues: GR) on leaf N, P, K, and Na contents (g kg^−1^) of chrysanthemum (*Chrysanthemum morifolium* cv. Pina colada) plants (OR: (**A1**–**A6**), GR: (**B1**–**B6**)). Bars indicate the mean ± SE (*n* = 6). Bars with the same letter do not differ significantly at *p ≥* 0.05 according to Duncan’s MRT.

Figure 2 presents the macronutrient content accumulated in chrysanthemum plant tissues, under OR- and GR-based substrate media cultivation. Plants grown using the OR-based substrates had lower N, P, Ca, and Mg accumulation compared to plants grown with CON treatment (Figure 2(A1–A6)). However, N content linearly increased with increasing levels of OR (Figure 2(A1)), while the highest OR level (40%) led to the highest accumulation of K (Figure 2(A3)), indicating a marginal contribution of OR to mineral accumulation, contingent on the volume used. In addition, Na content increased at OR ≥20%, compared to the rest of the treatments (Figure 2(A6)). In contrast, GR mixtures increased the content of N and K at GR ≥20% (Figure 2(B1,B3)). Additionally, GR 20% produced plants with the highest P accumulation (Figure (2B2)). In contrast, leaf Ca generally decreased with the addition of GR (Figure 2(B4)), while the lowest Mg content was found in plants grown with GR 5% (Figure 2(B5)). In the case of Na content, decreases occurred with the use of GR, with the lowest content being observed with the highest GR addition (Figure 2(B6)).

**Figure 3:**
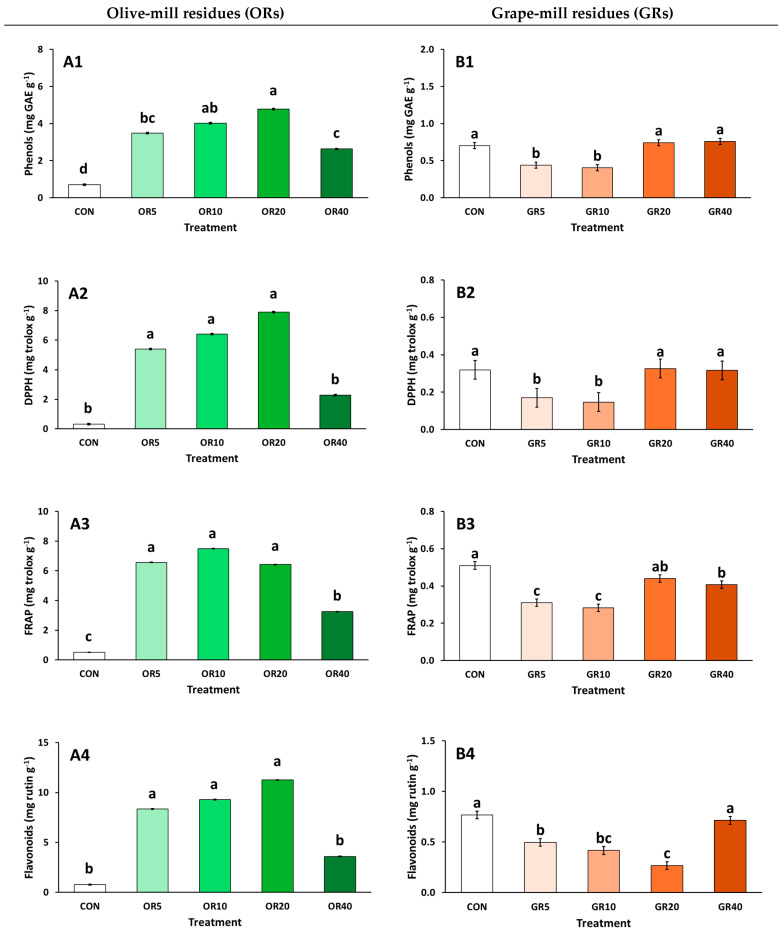
Impact of growing media (peat: CON; olive-mill residues: ORs; and grape-mill residues: GR) on the total phenols (mg GAE g^−1^ FW), antioxidant activity (DPPH, FRAP; mg trolox g^−1^ FW), and flavonoids (mg rutin g^−1^ FW) of chrysanthemum (*Chrysanthemum morifolium* cv. Pina colada) plants (OR: (**A1**–**A4**), GR: (**B1**–**B4**)). Bars indicate the mean ± SE (*n* = 6). Bars with the same letter do not differ significantly at *p ≥* 0.05 according to Duncan’s MRT.

The addition of OR and GR as partial substrate media affected the content of total phenols and flavonoids, as well as the antioxidant activity of chrysanthemum plants, as assayed by DPPH and FRAP (Figure 3). Total phenols were increased with the addition of OR, by up to 580% with the application of OR 20%, compared to CON (Figure 3(A1)). However, at OR 5% and OR 40%, total phenols were significantly lower than at OR 20%. Flavonoids and the antioxidant activity of chrysanthemum plants exhibited a similar response, with increases being observed in response to OR addition relative to CON (Figure 3(A2–A4)). However, the highest OR level (40%) resulted in a reduction in these biochemical parameters compared to lower OR levels, while no significant differences were detected for DPPH and flavonoid content, in comparison to CON. In the case of GR addition, chrysanthemum plants exhibited decreases in total phenolic content and antioxidant activity, at 5–10% GR addition, while at ≥20% GR, values remained at similar levels to CON (Figure 3(B1–B3)). However, flavonoid content remained at similar levels only with the highest GR addition, while decreases occurred at 5–20% GR (Figure 3(B4)).

**Figure 4:**
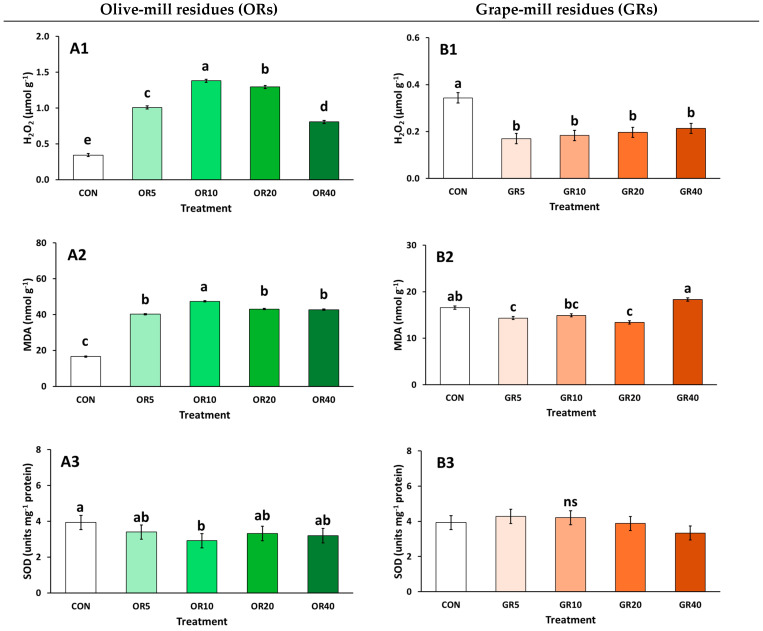

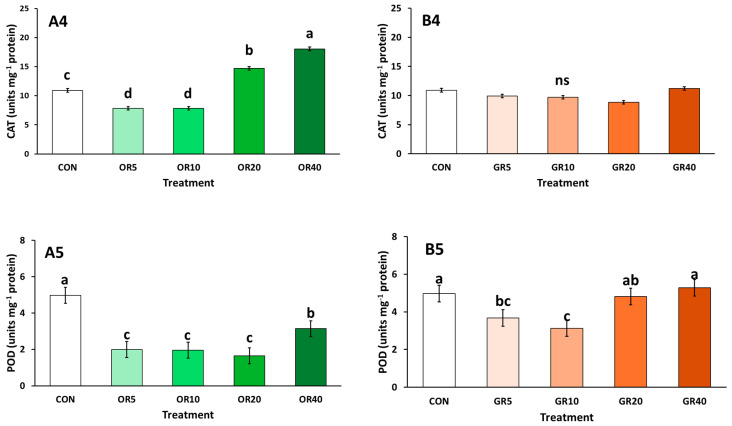
Impact of growing media (peat: CON; olive-mill residues: OR; and grape-mill residues: GR) on hydrogen peroxide (H_2_O_2_; μmol g^−1^ FW), lipid peroxidation (MDA; nmol g^−1^ FW), and antioxidant enzyme activity; superoxide dismutase (SOD), catalase (CAT), and peroxidase (POD) (units mg^−1^ protein) of chrysanthemum (*Chrysanthemum morifolium* cv. Pina colada) plants (OR: (**A1**–**A5**), GR: (**B1**–**B5**)). Bars indicate the mean ± SE (*n* = 6). Bars with the same letter do not differ significantly at *p ≥* 0.05 according to Duncan’s MRT.

Plants have a plethora of detoxification procedures during exposure to biotic and abiotic stress conditions to remove reactive oxygen species (ROS) generated in cells. Lipid peroxidation, which is linked to MDA generation, is a commonly evaluated stress marker. In the case of OR addition, increases occurred in chrysanthemum plants, compared to the use of CON (Figure 4(A2)). Stressful conditions with the application of OR are further indicated by the increase in H_2_O_2_ production (Figure 4(A1)), indicating cell damage, and is linked to the increase in the levels of phenols and antioxidants observed previously (Figure 3). CAT activity under these conditions is indicative of a slight reduction in H_2_O_2_, at OR 10% and OR 40% (Figure 4(A4)). Interestingly, increased POD activity is associated with the low levels of H_2_O_2_ and MDA recorded in plants grown using the CON treatment (Figure 4(A5)). Finally, H_2_O_2_ production was subdued with the highest OR level (40%), coupled with increased CAT compared to the rest of the treatments (Figure 4(A4)), and POD compared to other OR levels (Figure 4(A5)), indicating their activation towards the induced stress conditions.

In the case of GR addition, H_2_O_2_ production decreased with the presence of GR (Figure 4(B1)), while MDA content increased at the highest GR ratio used, i.e., 40% in the growing media, reaching similar levels as CON (Figure 4(B2)). POD activity followed the pattern of MDA production (Figure 4(B5)), while no differences were found for CAT and SOD enzyme activities among the treatments (Figure 4(B3,B4)).

## 3. Discussion

Not all materials are suitable for utilization as substrate media in containerized plant cultivation, or seedling production in nurseries. Factors such as pH, salinity, or the presence of phytochemicals (i.e., polyphenols) can cause phytotoxicity and stress, limiting their applicability [39,40]. Evaluating the suitability of wastes as horticultural substrates, and by extension, their impact on parameters such as plant growth and yield, is inherently complex, requiring a thorough examination of several physicochemical indices. The use of organic growing media, especially under soilless cultivation, affects nutrient uptake by plants due to the potential addition of nutrients available in the root zone. This should be considered when adjusting prescriptive fertilization regimes to avoid over-fertilization and excessive nutrient leaching [7]. Wastes derived from olive oil and wine production may contain substantial mineral levels; thus, their use should be carefully evaluated, especially in a raw form [41].

In the current study, the EC of the substrate mixtures containing OR and GR remained near or well below the maximum threshold for container media (≤0.5 mS cm^−1^), as reported by Abad et al. [42]. This is noteworthy because EC levels can limit the introduction of novel materials in the cultivation of plants sensitive to osmotic stress, including various ornamentals [7]. For chrysanthemum cultivation, an EC of 2.1 mS cm^−1^ in hydroponic NS is ideal for achieving market quality standards. However, in this study, no fertigation was applied, in order to isolate the effect of the culture media [43]. In the case of OR at ≥10%, significant increases in EC occurred, compared to CON. For GR, this was evident with the addition of GR ≥ 20%. These increases, proportional to the level of organic material used, have been previously reported [17]. However, EC ranged from 226.10 to 613.27 μS cm^−1^ for OR, and from 230.90 to 389.05 μS cm^−1^ for GR, levels generally considered low [44]. According to Greek standards, the EC of composted materials should not exceed 4 mS cm^−1^ [32]. The raw materials in this study had EC values well below this threshold, which is reflected in the prepared mixtures. Nonetheless, organic materials, including raw grape marc and compost, may have elevated EC levels that could potentially cause osmotic stress and ion imbalances [45].

The pH of the substrate mixtures was also varied. Generally, pH values should fall within a range of 5.5 to 6.5 [46]. The raw materials in the current study had a pH of 6.57 and 7.19, for OR and GR, respectively. The addition of GR, which had a higher pH than peat in its raw form, caused a progressive increase in the pH of the mixtures. This suggests that GR could potentially be used with peat without the need to add lime for pH corrections, which is a common practice. However, the pH values attained at 5–10% GR (6.5–6.9) are not considered limiting in soilless cultivation, as they are close to the optimal range of 5.5–6.5. Moreover, a pH of up to 7.0 may not pose significant issues for most crops [47]. Conversely, the incorporation of OR did not cause significant deviations in pH compared to the CON treatment, as the raw OR material had a pH value similar to that of limed peat. In soilless substrate cultivation, lower pH and EC values are generally preferred for the preparation and production of growing media [47].

Moreover, the physical properties of soilless growing media have a pronounced impact on the overall growth, as they influence air and water availability [47]. The substrate mixtures were evaluated according to the acceptable/optimum ranges for selected physical properties, as defined by Abad et al. [42]. Specifically, total porosity (TP) and available water-holding capacity (AWHC) were close to the reference values in both OR and GR mixtures. In addition, bulk density (BD) was below the maximum value of 0.4 g cm^−3^ for both materials, although their introduction significantly increased it. However, air-filled porosity (AFP) was below the recommended levels, although the highest GR level marginally increased it compared to CON. This low value has been previously observed [48].

Organic matter content ranged from 93.63 to 94.87% for OR mixtures and from 93.06 to 93.18% for GR mixtures. Although GR had a lower organic matter content compared to CON, all OR and GR mixtures exhibited increased values, as similarly observed by Chrysargyris et al. [49]. Prior to planting, mixtures had elevated levels of minerals. Specifically, mixtures containing OR and GR resulted in an increase in N, compared to CON. However, OR was primarily associated with reduced levels of P, Ca, Mg, and Na. In contrast, mixtures containing GR had higher mineral levels; its addition led to a significant increase in N, P, and K contents, compared to CON. Following chrysanthemum cultivation, mineral contents did not significantly deviate from initial values, although N levels increased at the highest OR concentration. As with other raw, uncomposted materials, their incorporation must be carefully regulated due to the absence of prior decomposition [50,51]. The excessive accumulation of minerals may lead to mineral imbalances, through mineral antagonism and interactions that disrupt plant growth and development. In OR mixtures, the decline in Mg levels coincided with an increase in K levels, consistent with the well-reported phenomenon of potassium-induced Mg deficiency in agricultural production [52].

As previously stated, the physiochemical properties of the growing media have a direct influence on plant growth. This is evident in the results of the current study, where the addition of OR led to decreased growth indices (e.g., plant height, leaf number, FW, DW, flower number) compared to CON, with more severe effects at higher OR percentages. Similarly, reductions in purslane (*Portulaca oleracea*) plant height, leaf number, and biomass production were observed with the addition of OR in peat-based substrate mixtures, especially beyond 10% OR [32]. However, Papafotiou et al. [53] observed minimal impact on the growth of *Syngonium podophyllum* at up 25% OR, and *Codiaeum veriegatum* at up to 75% OR. These results highlight the interactions among OR composition, prior composting processes, application rates, and the plant species involved. In contrast, GR behaved similarly at GR 20% and better at 5–10% GR, compared to CON, exhibiting stable or improved plant height, shoot diameter, and biomass production. Flower production also remained stable, at up to 20% GR. Similarly, in the soilless cultivation of carnation (*Dianthus caryophyllus*), plant growth remained unaffected with the addition of 5–10% GR, although a reduction in the leaf number occurred at 20% GR [49]. In addition, the use of grapevine and grape stalk compost in gerbera cultivation did not negatively affect the flower number of gerbera (*Gerbera jamesonii*), compared to commercial peat substrate cultivation [22].

In plant physiology, chlorophyll fluorescence decreased with OR application, compared to CON. Reduced Fv/Fm levels indicate plant stress, as impaired PSII reaction centers hinder electron transport efficiency [54]. Similarly, a decline in relative chlorophyll content, as measured by SPAD, directly affects photosynthesis and contributes to overall plant growth reduction under OR applications. Stress was also evident through reduced stomatal conductance, as plants respond to stress by closing their stomata [32]. The unfavorable conditions introduced by OR in the substrate media, as well as their direct impact on plant physiology, have been previously reported [32]. However, in this experiment, plants received no fertigation. Studies suggest that proper mineral application may mitigate issues related to reduced chlorophyll content [33]. The stress response may also stem from the unfavorable physical properties of the growing media containing OR, such as TP and AFP. To improve conditions, amendments with inert materials like perlite and vermiculite should be considered. In contrast, the addition of GR had minimal effects on plant physiology, except with the applications of ≥20% GR. Similarly, Chrysargyris et al. [49] found no significant effects on Fv/Fm and SPAD when incorporating up to 20% GR in peat-based substrates for carnation cultivation. This might be attributed to the presence and availability of nutrients, especially nitrogen, as revealed by the mineral analysis of the plant tissue as well. However, Carmona et al. [22] reported slight decreases in SPAD values after adding grapevine marc and grape stalk compost. These findings highlight the importance of the initial material composition, physical properties, and species-specific responses.

Organic materials like OR and GR contain substantial mineral content, making composting a viable method for recycling these residues [17,55]. Mineral uptake by plants depends on the physicochemical properties of the substrate, including nutrient release dynamics, pH, salinity, and physical features such as water retention and aeriation [46,56,57]. The underperforming residue, OR, resulted in plants with lower N, P, Ca, and Mg levels in leaves. Reduced N accumulation may be due to its limited availability, negatively impacting plant growth, chlorophyll accumulation, and photosynthesis [41,58]. The reduced Mg levels, found both in OR mixtures and in plant tissue, might explain the poor performance of plants under OR. Mg deficiency restricts crop growth and the distribution of photosynthetic products, leading to severe declines in crop yield and quality [59]. Additionally, nutrient uptake variations across substrates are influenced by overall physicochemical properties and potential stress from phytotoxic substances like phenols [60,61]. These phenolic compounds may not only be responsible for phytotoxicity, but may also modify the substrate microflora, significantly affecting the C/N ratio and potentially releasing phytotoxins through increased microbial activity [62,63].

In contrast, chrysanthemum plants exhibited optimal growth under GR applications, as reflected in their nutritional status. Increasing GR levels corresponded with higher N accumulation, likely due to the elevated N content in the substrate. Leaf P and levels also increased with GR application compared to CON, depending on the percentage used. However, Ca levels declined at GR 5, 20, and 40%, while Mg content decreased at GR 5%. Similar findings were observed by Bustamante et al. [64], where composts derived from distillery waste provided adequate nutrition to various seedlings (*Lactuca sativa*, *Beta vulgaris*, *Brassica oleracea*, *Coriandrum sativum*). They noticed that the increasing ratio of such materials in the substrates enhanced macronutrient content, with these increments being proportional to the addition, and the opposite occurred for Mg. However, due to the variability in the mineral composition and release rates of raw materials, appropriate fertigation could help ensure standardized and optimal plant nutrition, minimizing fluctuations in nutrient availability [49].

The different substrate mixtures influenced the total phenolic content, antioxidants, and flavonoids in chrysanthemum plants. Notably, OR incorporation led to an increase in all measured parameters, suggesting enhanced secondary metabolite accumulation. Chrysargyris et al. [32] previously observed a similar effect in soilless purslane cultivation with OR, which significantly increased total phenolics and antioxidants, as the plant’s first metabolic response to a new/different condition that it has to adapt to. This response indicates stress conditions, likely due to OR’s poor performance as a substrate component, as evidenced by elevated H_2_O_2_ and MDA levels. Under stress, plants generate reactive oxygen species (ROS), triggering antioxidant enzyme activity to mitigate oxidative damage [65,66]. SOD activity detoxifies ROS, followed by CAT and POD, which break down H_2_O_2_ [32]. However, in plants grown with OR, SOD and CAT activity remained unchanged or decreased, compared to CON, with CAT activity increasing only at high OR rates (≥20% OR). This, alongside elevated H_2_O_2_ and MDA, suggests that chrysanthemum plants struggled to manage stress under the OR-amended conditions. A similar trend was observed by Chrysargyris et al. [67], where OR in the substrate increases lipid peroxidation and H_2_O_2_ production in *Brassica* seedlings. This stress response may stem from phytotoxic compounds in the raw OR, such as phenols and organic acids. Composting OR can significantly reduce phytotoxicity by degrading these toxic compounds, creating a more stable material [19,61].

In contrast, GR addition did significantly increase plant stress, as H_2_O_2_ and MDA levels remained stable. At lower GR levels (5–10%), total phenolics, antioxidant activity, and flavonoids decreased. Given the enhanced plant growth parameters observed in treatments containing GR, compared to control plants grown in 100% peat, it is evident that the physicochemical properties of the GR mixtures (5–10%) created optimal growing conditions for chrysanthemum plants, without causing any physiological disturbance or stress conditions. However, at higher GR levels (20–40%), total phenolics and antioxidants increased, despite relatively stable H_2_O_2_ and antioxidant enzyme activity compared to CON. Nonetheless, at 40% GR, MDA levels rose, flower production declined, suggesting this mixture may not be commercially viable for chrysanthemum cultivation. Similarly, a study incorporating up to 20% GR in peat-based substrate found no signs of stress in carnation plants, as H_2_O_2_ and MDA levels remained unchanged compared to CON (pure peat), while antioxidant activity and phenol content also remained unchanged [49]. These results suggest the suitability of the mixtures with GR up to a certain degree for the growth and production of potted ornamental plants.

## 4. Materials and Methods

### 4.1. Preparation of Plant Material and Substrate Mixtures

The current experiment was performed in the research hydroponic greenhouse of Cyprus University of Technology, located in Limassol, Cyprus, at 34.700120°N, 32.984276°E. Commercial peat (professional-grade peat, Rėkyva JSC, Šiauliai, Lithuania) served as the control media (CON). Olive-mill residues (ORs) were obtained from a local olive oil production company (Limassol, Cyprus), produced by multi-variety olive oil extraction. Grape-mill (winery) residues (GRs) were obtained after the production of red wine of the “Mavro” variety, an indigenous variety to Cyprus, from a local winery (Limassol, Cyprus). The properties of the examined residues are displayed in Table 4. The prepared substrate mixtures contained peat, and a gradual substitution of peat at different ratios (by volume) of OR and GR materials resulted in the following mixtures (*v*/*v*): (1) Peat/OR; 100:0 (CON, served as control), (2) Peat/OR; 95:5 (OR 5%), (3) Peat/OR; 90:10 (OR 10%), (4) Peat/OR; 80:20 (OR 20%), and (5) Peat/OR; 60:40 (OR 40%) for olive-mill residues, and (6) Peat/GR; 100:0 (CON served as control), (7) Peat/GR; 95:5 (GR 5%), (8) Peat/GR; 90:10 (GR 10%), (9) Peat/GR; 80:20 (GR 20%), and (10) Peat/GR; 60:40 (GR 40%) for grape-mill residues. Before plant transplanting, samples from each substrate mixture were collected for the determination of their physical, chemical, and hydrological properties. The mixtures were established based on prior research on residue incorporation in growing media [32].

Rooted cuttings of chrysanthemum (*C. morifolium* cv. Pina Colada), obtained from a local nursery, were transplanted to 2 L pots, containing the nine different mixtures. For each mixture, six replicate pots were used, each one accommodating a single plant. Pots were positioned in plates to retain drainage after irrigation. Plants were irrigated individually, according to their growth and needs. During cultivation, no fertilizers and agrochemicals were applied. The temperature during the experimental phase averaged 22.52 °C, while air humidity averaged 55.50%.

### 4.2. Substrate Mixture Properties

The raw residues and initial substrate mixtures were examined to determine various properties, including total porosity (TP; % *v*/*v*), available water-holding capacity (AWHC; % *v*/*v*), air-filled porosity (AFP; % *v*/*v*), and bulk density (BD; g cm^−3^) under the European Standard EN 12,041 described previously [33]. For raw materials and both initial and final substrates, measurements of pH and EC were conducted using the 1:5 (*v*/*v*) method, while organic matter content was calculated following furnace ashing at 550 °C [68]. The ashes were subsequently acid-digested for mineral analysis. Flame photometry was utilized for the determination of potassium (K) and sodium (Na), with the Lasany Model 1832 flame photometer (Lasany International, Pachkula, India). Phosphorus (P) was determined based on molybdate/vanadate (yellow method). The determination of nitrogen (N) was performed according to the Kjeldahl method (BUCHI, Digest automat K-439 and Distillation Kjelflex K-360, Flawil, Switzerland). Finally, magnesium (Mg) and calcium (Ca) were determined using an atomic absorption spectrophotometer (PG Instruments AA500FG, Leicestershire, UK). The macronutrient content is expressed in g kg^−1^ DW.

### 4.3. Plant Growth, Physiology, and Mineral Analysis

Chrysanthemum plants were grown for a total of 44 days after transplanting (DAT). Afterwards, several growth-related parameters were evaluated, including the number of leaves, stems, and flowers (closed and opened), as well as the plants’ height and main stem thickness. Plants were then harvested, and the upper biomass production (Fresh weight-FW; g) was measured. Tissues were subsequently dried at 42 °C for 96 h, in an air-ventilated oven, and the dry weight (DW; g) was used to determine the leaves’ dry matter content (DMC; %).

Various non-destructive physiological measurements were conducted before harvesting. Leaf chlorophyll fluorescence (Fv/Fm) was recorded on six fully expanded leaves per treatment (Opti-Sciences fluorometer OS-30p, Hertfordshire, UK). Relative chlorophyll content was determined by soil–plant analysis development (SPAD) using the SPAD-502 Plus (Konica Minolta, Tokyo, Japan). Stomatal conductance determination was undertaken with a ΔT-Porometer (AP4, Delta-T Devices, Cambridge, UK).

Plant macronutrient content was determined on three replications per treatment. Dried plant tissue was ashed and acid-digested using 2 N HCl [69]. Afterwards, K and Na were determined by flame photometry (Lasany Model 1832, Lasany International, Pachkula, India), P by the molybdate vanadate method, and N by the Kjeldahl method (BUCHI, Digest automat K-439 and Distillation Kjelflex K-360, Flawil, Switzerland). Finally, Mg and Ca were determined by atomic absorption spectrophotometry (PG Instruments AA500FG, Leicestershire, UK). The macronutrient content is expressed in g kg^−1^ DW.

### 4.4. Total Phenolics, Total Flavonoids, and Antioxidant Activity

Methanolic extracts of leaf tissues were prepared to determine the content of total phenolics, flavonoids, and antioxidant activity, as assayed previously [33]. Total phenols were determined by employing the Folin–Ciocalteu method, with measurements of absorbance at 755 nm [33]. Values are expressed in gallic acid equivalents (mg GA g^−1^ FW).

Flavonoid determination was conducted by a modified aluminum chloride colorimetric assay, with absorbance measurements at 510 nm [33]. Values are expressed in rutin equivalents (mg rutin g^−1^ FW).

Free radical scavenging activity was determined using DPPH (2,2-diphenyl-1-picrylhydrazyl) measurements at 517 nm, and FRAP (ferric-reducing antioxidant power) measurements at 593 nm [33]. In all protocols, a Trolox (( ± )-6-hydroxy-2,5,7,8-tetramethylchromane-2-carboxylic acid) positive control was utilized, and the values are expressed in Trolox equivalents (mg Trolox g^−1^ FW).

### 4.5. Stress Indicators and Antioxidant Enzyme Activity

The content of hydrogen peroxide (H_2_O_2_) was assayed as described by Loreto and Velikova [70] with measurements at 390 nm and expressed as μmol H_2_O_2_ g^−1^ FW. Lipid peroxidation–malondialdehyde (MDA) content was assessed as previously reported [71], with measurements at 532 nm, corrected for non-specific absorbance (600 nm). MDA content was measured by using the extinction coefficient of 155 mM cm^−1^ and expressed as nmol MDA g^−1^ FW.

Antioxidant activity for superoxide dismutase (SOD) (EC 1.15.1.1) and catalase (CAT) (EC 1.11.1.6) was evaluated as previously described [32], by measuring the absorbance at 560 and 240 nm, respectively. Peroxidase (POD) activity (EC 1.11.1.6) was measured by following the increase in absorbance at 430 nm, as previously assessed [32]. The results for SOD, CAT, and POD were expressed as enzyme units mg^−1^ protein. Protein content assessment in leaves was conducted using the Bradford method and bovine serum albumin as a standard.

### 4.6. Statistical Analysis

Analysis of variance (ANOVA) was applied using IBM SPSS v.22 to evaluate the effect of different substrate mixtures on chrysanthemum growth, physiology, and stress response. Data were analyzed as treatment mean  ±  standard error (SE). Mean values represent three replicates for substrate physiological properties, fresh weight, mineral determination, stomatal conductance, total phenolics, total flavonoids, antioxidant activity, lipid peroxidation, hydrogen peroxide, and the activity of the antioxidant enzymes; and six replicates for plant growth indices, as well as physiological and biochemical parameters. Duncan’s multiple range tests were performed when ANOVA indicated a significant treatment impact at *p* < 0.05.

## 5. Conclusions

Peat, a key component of soilless growing media, has a high carbon footprint, posing a challenge in finding suitable peat alternatives that maintain fertility and physicochemical properties while reducing environmental impact. This study partially replaced peat with residues from olive oil (OR) and wine (GR) for containerized chrysanthemum cultivation.

The incorporation of these materials influenced the physicochemical properties of the substrate mixtures. Both OR and GR mixtures had an EC close to or below the maximum acceptable level for container media (≤0.5 mS cm^−1^). GR significantly increased pH due to its raw material’s naturally higher pH (7.19), but at 5–10% incorporation, it remained within viable limits for soilless cultivation. OR reduced total porosity, limiting water retention, whereas GR increased porosity at 20–40% GR, due to its high natural porosity. Bulk density increased with both materials but remained below standard thresholds.

OR additions negatively affected chrysanthemum growth, reducing flower production, chlorophyll fluorescence, and relative chlorophyll content, with severity depending on application levels. Plants grown in OR-containing mixtures exhibited lower leaf N, P, Ca, and Mg levels, while stress indicators, including total phenolics, total flavonoids, antioxidant activity, antioxidant enzymes, and MDA and H_2_O_2_ levels, were elevated. This suggests that OR reduced oxidative stress. In contrast, GR did not cause significant oxidative stress or impair plant growth, physiology, or nutrient uptake. However, at 40% GR, the flower production declined, and MDA levels increased. Based on these findings, GR can be effectively used as a peat substitute up to 20% in soilless chrysanthemum cultivation, as plants demonstrated comparable or enhanced growth compared to those in pure peat. Conversely, OR exhibited suboptimal performance, requiring strategies for improved integration into soilless cultivation. These may include composting for phytotoxicity reduction, optimizing application volumes, and combining residues like OR and GR to enhance substrate properties.

Further research should investigate the effects of composted materials on plant growth, particularly soilless systems and crop nutrition. Additionally, studies should assess the direct application of raw agricultural residues in field and container cultivation. Finally, combining innovative strategies for the sustainable reuse of agricultural waste will be essential in promoting a circular and environmentally friendly agricultural system.

## Figures and Tables

**Table 1:** Physicochemical properties of growing media (peat: CON; olive-mill residues: OR; and grape-mill residues: GR) before chrysanthemum (*Chrysanthemum morifolium* cv. Pina Colada) transplanting.

	**CON**	**OR 5%**	**OR 10%**	**OR 20%**	**OR 40%**
pH	6.44 ± 0.05 b	6.69 ± 0.01 a	6.47 ± 0.01 b	6.51 ± 0.01 b	6.74 ± 0.03 a
EC (μS cm^−1^)	210.00 ± 16.26 d	226.10 ± 6.93 d	290.78 ± 15.54 c	350.40 ± 24.65 b	613.27 ± 2.98 a
Organic matter (%)	94.29 ± 0.17 a	93.63 ± 0.06 b	94.87 ± 0.33 a	94.57 ± 0.12 a	94.66 ± 0.21 a
Organic C (%)	54.70 ± 0.10 a	54.31 ± 0.03 b	55.03 ± 0.19 a	54.85 ± 0.07 a	54.91 ± 0.12 a
C/N ratio	81.14 ± 1.27 a	73.53 ± 2.71 b	71.38 ± 0.24 b	71.92 ± 1.63 b	72.19 ± 2.55 b
N%	0.68 ± 0.01 b	0.74 ± 0.03 ab	0.77 ± 0.00 a	0.76 ± 0.02 a	0.76 ± 0.03 a
N (g kg^−1^)	6.75 ± 0.10 b	7.41 ± 0.28 a	7.71 ± 0.00 a	7.64 ± 0.18 a	7.63 ± 0.28 a
P (g kg^−1^)	0.93 ± 0.01 a	0.84 ± 0.01 b	0.82 ± 0.03 b	0.78 ± 0.01 b	0.63 ± 0.03 c
K (g kg^−1^)	1.51 ± 0.09 d	2.41 ± 0.07 c	2.74 ± 0.02 c	3.43 ± 0.04 b	4.01 ± 0.28 a
Ca (g kg^−1^)	20.95 ± 0.37 a	17.97 ± 0.42 b	15.67 ± 0.15 c	13.73 ± 0.29 d	9.26 ± 0.27 e
Mg (g kg^−1^)	2.02 ± 0.03 a	1.71 ± 0.05 b	1.56 ± 0.02 c	1.40 ± 0.04 d	0.96 ± 0.05 e
Na (g kg^−1^)	0.34 ± 0.00 a	0.32 ± 0.01 ab	0.30 ± 0.01 b	0.31 ± 0.00 b	0.27 ± 0.01 c
Total porosity (% *v*/*v*)	82.73 ± 0.96 a	74.71 ± 0.19 b	81.74 ± 3.06 ab	75.84 ± 2.99 ab	74.92 ± 2.69 b
Air-filled porosity (% *v*/*v*)	12.88 ± 0.83 a	8.93 ± 1.03 b	11.43 ± 1.03 a	6.61 ± 0.10 bc	5.36 ± 0.21 c
Bulk density (g cm^−3^)	0.18 ± 0.01 d	0.18 ± 0.00 d	0.22 ± 0.01 c	0.24 ± 0.00 b	0.35 ± 0.01 a
Container capacity (% *v*/*v*)	69.85 ± 0.17 a	65.78 ± 1.22 a	70.32 ± 2.03 a	69.24 ± 3.09 a	69.55 ± 2.90 a
	**CON**	**GR 5%**	**GR 10%**	**GR 20%**	**GR 40%**
pH	6.44 ± 0.05 e	6.60 ± 0.06 d	6.90 ± 0.05 c	7.10 ± 0.05 b	7.41 ± 0.01 a
EC (μS cm^−1^)	210.00 ± 16.26 b	230.90 ± 22.11 b	265.59 ± 16.72 b	351.65 ± 43.39 a	389.05 ± 1.90 a
Organic matter (%)	94.29 ± 0.17 a	93.16 ± 0.24 b	93.06 ± 0.16 b	93.18 ± 0.25 b	93.06 ± 0.31 b
Organic C (%)	54.70 ± 0.10 a	54.03 ± 0.14 b	53.98 ± 0.09 b	54.05 ± 0.15 b	53.98 ± 0.18 b
C/N ratio	81.14 ± 1.27 a	60.18 ± 0.91 b	51.78 ± 1.07 c	36.52 ± 0.75 d	29.54 ± 1.08 e
N%	0.68 ± 0.01 e	0.90 ± 0.01 d	1.04 ± 0.02 c	1.48 ± 0.03 b	1.83 ± 0.07 a
N (g kg^−1^)	6.75 ± 0.10 e	8.98 ± 0.11 d	10.43 ± 0.22 c	14.81 ± 0.27 b	18.33 ± 0.72 a
P (g kg^−1^)	0.93 ± 0.01 d	1.36 ± 0.05 c	1.26 ± 0.01 c	1.57 ± 0.09 b	2.10 ± 0.06 a
K (g kg^−1^)	1.51 ± 0.09 e	3.56 ± 0.03 d	4.57 ± 0.05 c	5.79 ± 0.06 b	6.76 ± 0.15 a
Ca (g kg^−1^)	20.95 ± 0.37 a	21.98 ± 0.65 a	18.16 ± 0.59 b	16.76 ± 0.61 b	13.59 ± 0.26 c
Mg (g kg^−1^)	2.02 ± 0.03 b	2.22 ± 0.09 a	1.83 ± 0.06 c	1.78 ± 0.04 c	1.54 ± 0.03 d
Na (g kg^−1^)	0.34 ± 0.00 a	0.34 ± 0.00 a	0.31 ± 0.00 b	0.27 ± 0.01 c	0.21 ± 0.01 d
Total porosity (% *v*/*v*)	82.73 ± 0.96 b	79.60 ± 1.72 bc	77.83 ± 1.56 bc	75.88 ± 0.89 c	87.96 ± 2.05 a
Air-filled porosity (% *v*/*v*)	12.88 ± 0.83 b	9.30 ± 0.82 c	8.75 ± 0.10 c	14.11 ± 0.31 ab	15.03 ± 0.21 a
Bulk density (g cm^−3^)	0.18 ± 0.01 c	0.19 ± 0.00 c	0.19 ± 0.00 c	0.21 ± 0.01 b	0.24 ± 0.01 a
Container capacity (% *v*/*v*)	69.85 ± 0.17 a	70.29 ± 0.89 a	69.08 ± 1.67 a	61.77 ± 0.59 b	72.93 ± 2.25 a

Total porosity (TP), air-filled porosity (AFP), bulk density (BD), and available water-holding capacity (AWHC—container capacity) by volume. Values are the mean ± SE (*n* = 6). In each row, values followed by the same letter for the different materials (OR, GR) are not significantly different, *p <* 0.05.

**Table 2:** Physicochemical properties of growing media (peat: CON; olive-mill residues: OR; and grape-mill residues: GR) after chrysanthemum (*Chrysanthemum morifolium* cv. Pina Colada) cultivation.

	**CON**	**OR 5%**	**OR 10%**	**OR 20%**	**OR 40%**
pH	6.49 ± 0.06 e	6.74 ± 0.02 d	6.97 ± 0.01 c	7.28 ± 0.02 b	7.43 ± 0.03 a
EC (μS cm^−1^)	541.40 ± 22.82 b	670.95 ± 48.93 a	225.70 ± 33.37 d	358.85 ± 2.68 c	608.06 ± 9.95 ab
Organic matter (%)	92.63 ± 0.30 a	91.39 ± 0.30 ab	94.13 ± 0.11 a	88.03 ± 2.64 b	95.21 ± 0.21 a
Organic C (%)	53.73 ± 0.18 a	53.01 ± 0.17 ab	54.60 ± 0.06 a	51.06 ± 1.53 b	55.22 ± 0.12 a
C/N ratio	88.29 ± 1.74 a	76.71 ± 2.01 b	75.72 ± 1.94 b	66.28 ± 2.16 c	62.29 ± 0.82 c
N%	0.61 ± 0.01 d	0.69 ± 0.02 c	0.72 ± 0.02 bc	0.77 ± 0.00 b	0.89 ± 0.01 a
N (g kg^−1^)	6.09 ± 0.14 d	6.92 ± 0.20 c	7.22 ± 0.19 c	7.70 ± 0.02 b	8.87 ± 0.13 a
P (g kg^−1^)	0.58 ± 0.00 b	1.43 ± 0.09 a	0.51 ± 0.05 b	0.69 ± 0.13 b	0.62 ± 0.01 b
K (g kg^−1^)	1.01 ± 0.01 e	2.92 ± 0.04 b	1.38 ± 0.10 d	1.91 ± 0.01 c	4.38 ± 0.07 a
Mg (g kg^−1^)	2.38 ± 0.05 a	2.34 ± 0.04 a	1.96 ± 0.01 b	1.35 ± 0.20 c	1.20 ± 0.03 c
Ca (g kg^−1^)	20.31 ± 0.51 a	20.63 ± 0.59 a	16.24 ± 0.13 b	11.58 ± 1.84 c	9.90 ± 0.21 c
Na (g kg^−1^)	4.19 ± 0.06 a	3.90 ± 0.05 b	2.27 ± 0.09 c	1.70 ± 0.11 d	1.28 ± 0.03 e
	**CON**	**GR 5%**	**GR 10%**	**GR 20%**	**GR 40%**
pH	6.49 ± 0.06 d	6.77 ± 0.01 c	7.32 ± 0.01 b	7.40 ± 0.01 b	7.53 ± 0.04 a
EC (μS cm^−1^)	541.40 ± 22.82 ab	633.70 ± 13.97 a	314.05 ± 47.37 c	461.75 ± 27.74 b	488.06 ± 24.23 b
Organic matter (%)	92.63 ± 0.30 a	91.96 ± 0.21 b	92.92 ± 0.05 a	92.63 ± 0.16 a	92.77 ± 0.17 a
Organic C (%)	53.73 ± 0.18 a	53.34 ± 0.12 b	53.90 ± 0.03 a	53.73 ± 0.09 a	53.81 ± 0.10 a
C/N ratio	88.29 ± 1.74 a	72.09 ± 0.41 b	55.21 ± 2.25 c	42.59 ± 0.36 d	32.97 ± 0.14 e
N%	0.61 ± 0.01 e	0.74 ± 0.01 d	0.98 ± 0.04 c	1.26 ± 0.01 b	1.63 ± 0.01 a
N (g kg^−1^)	6.09 ± 0.14 e	7.40 ± 0.06 d	9.80 ± 0.40 c	12.62 ± 0.09 b	16.32 ± 0.07 a
P (g kg^−1^)	0.58 ± 0.00 d	0.99 ± 0.12 bc	0.82 ± 0.04 c	1.20 ± 0.01 ab	1.38 ± 0.09 a
K (g kg^−1^)	1.01 ± 0.01 e	2.08 ± 0.08 d	2.44 ± 0.09 c	3.91 ± 0.08 b	6.13 ± 0.15 a
Mg (g kg^−1^)	2.38 ± 0.05 ab	2.51 ± 0.05 ab	2.57 ± 0.19 a	2.45 ± 0.16 ab	2.16 ± 0.04 b
Ca (g kg^−1^)	20.31 ± 0.51 a	20.38 ± 0.11 a	18.89 ± 0.26 a	19.93 ± 1.00 a	15.13 ± 0.62 b
Na (g kg^−1^)	4.19 ± 0.06 a	4.35 ± 0.12 a	3.79 ± 0.04 b	2.94 ± 0.05 c	2.45 ± 0.02 d

Total porosity (TP), air-filled porosity (AFP), bulk density (BD), and available water-holding capacity (AWHC—container capacity) by volume. Values are the mean ± SE (*n* = 6). In each row, values followed by the same letter for the different materials (OR, GR) are not significantly different, *p <* 0.05.

**Table 3:** Impact of growing media (peat: CON; olive-mill residues: OR; and grape-mill residues: GR) on chrysanthemum (*Chrysanthemum morifolium* cv. Pina Colada) plant growth and flower production.

	**CON**	**OR 5%**	**OR 10%**	**OR 20%**	**OR 40%**
Plant height (cm)	30.27 ± 2.26 a	23.17 ± 1.59 b	19.42 ± 0.67 bc	18.27 ± 0.56 c	13.20 ± 0.76 d
Shoot diameter (mm)	4.34 ± 0.18 a	3.29 ± 0.09 b	3.37 ± 0.16 b	3.07 ± 0.13 b	2.44 ± 0.17 c
Leaf number	23.50 ± 3.58 a	14.50 ± 0.56 b	13.00 ± 0.86 b	11.33 ± 0.56 b	10.33 ± 0.80 b
Lateral shoot number	n.m.	n.m.	n.m.	n.m.	n.m.
Plant FW (g)	25.18 ± 4.44 a	6.70 ± 0.65 b	4.83 ± 0.49 b	3.98 ± 0.50 b	3.48 ± 0.35 b
Leaf FW (g)	8.77 ± 2.63 a	3.94 ± 0.30 b	2.77 ± 0.42 b	3.16 ± 0.39 b	2.66 ± 0.23 b
Shoot FW (g)	9.17 ± 2.22 a	2.39 ± 0.29 b	1.63 ± 0.16 b	1.45 ± 0.24 b	1.02 ± 0.15 b
Flower FW (g)	10.87 ± 2.76 a	0.92 ± 0.14 b	0.62 ± 0.18 b	0.49 ± 0.19 b	0.35 ± 0.09 b
Plant DW (g)	4.70 ± 1.50 a	1.88 ± 0.20 b	1.28 ± 0.19 b	1.32 ± 0.19 b	0.83 ± 0.08 b
Leaf DW (g)	0.99 ± 0.34 a	0.90 ± 0.08 a	0.62 ± 0.11 a	0.72 ± 0.07 a	0.45 ± 0.02 a
Shoot DW (g)	2.32 ± 0.76 a	0.83 ± 0.10 b	0.57 ± 0.07 b	0.52 ± 0.09 b	0.33 ± 0.04 b
Flower DW (g)	1.39 ± 0.40 a	0.16 ± 0.02 b	0.09 ± 0.02 b	0.08 ± 0.03 b	0.06 ± 0.01 b
Plant DM (%)	15.52 ± 0.94 d	25.45 ± 0.70 b	27.40 ± 0.99 ab	29.38 ± 1.62 a	20.54 ± 0.62 c
Open flower number	3.67 ± 1.17 a	0.00 ± 0.00 b	0.00 ± 0.00 b	0.00 ± 0.00 b	0.00 ± 0.00 b
Closed flower number	6.33 ± 0.61 a	3.50 ± 0.34 b	2.83 ± 0.31 b	1.33 ± 0.21 c	1.17 ± 0.17 c
Total flower number	10.00 ± 0.93 a	3.50 ± 0.34 b	2.83 ± 0.31 b	1.33 ± 0.21 c	1.17 ± 0.17 c
	**CON**	**GR 5%**	**GR 10%**	**GR 20%**	**GR 40%**
Plant height (cm)	30.27 ± 2.26 ab	34.88 ± 3.37 a	37.48 ± 3.55 a	25.24 ± 4.04 b	14.75 ± 0.64 c
Shoot diameter (mm)	4.34 ± 0.18 ab	4.91 ± 0.37 a	5.04 ± 0.39 a	3.67 ± 0.17 bc	3.06 ± 0.17 c
Leaf number	23.50 ± 3.58 b	23.17 ± 2.34 b	35.80 ± 7.45 a	21.33 ± 3.33 b	11.50 ± 0.62 b
Lateral shoot number	2.83 ± 0.70 bc	6.50 ± 1.23 a	5.60 ± 1.25 ab	2.60 ± 0.68 bc	2.00 ± 0.58 c
Plant FW (g)	25.18 ± 4.44 b	49.20 ± 10.78 a	56.50 ± 16.31 a	14.55 ± 1.95 b	5.92 ± 0.80 b
Leaf FW (g)	8.77 ± 2.63 b	24.13 ± 1.94 a	17.43 ± 2.97 a	8.28 ± 1.51 b	4.87 ± 0.34 b
Shoot FW (g)	9.17 ± 2.22 b	22.49 ± 2.01 a	18.12 ± 2.97 a	7.37 ± 1.81 bc	1.23 ± 0.03 c
Flower FW (g)	10.87 ± 2.76 ab	19.07 ± 3.04 a	15.78 ± 4.17 ab	7.30 ± 2.03 bc	0.40 ± 0.00 c
Plant DW (g)	4.70 ± 1.50 bc	11.67 ± 1.20 a	8.32 ± 1.89 ab	3.43 ± 0.81 c	0.89 ± 0.07 c
Leaf DW (g)	0.99 ± 0.34 c	2.72 ± 0.22 a	1.78 ± 0.32 b	0.87 ± 0.13 c	0.55 ± 0.06 c
Shoot DW (g)	2.32 ± 0.76 bc	6.31 ± 0.64 a	4.48 ± 1.01 ab	1.62 ± 0.43 c	0.28 ± 0.01 c
Flower DW (g)	1.39 ± 0.40 ab	2.64 ± 0.40 a	2.05 ± 0.55 ab	0.94 ± 0.26 bc	0.06 ± 0.01 c
Plant DM (%)	15.52 ± 0.94 ab	17.19 ± 0.44 a	15.19 ± 0.58 ab	14.46 ± 0.18 bc	12.91 ± 1.02 c
Open flower number	3.67 ± 1.17 a	4.00 ± 1.44 a	4.00 ± 2.05 a	3.25 ± 2.25 a	0.00 ± 0.00 b
Closed flower number	6.33 ± 0.61 a	8.33 ± 1.12 a	9.33 ± 1.36 a	7.33 ± 2.42 a	1.17 ± 0.17 b
Total flower number	10.00 ± 0.93 a	12.33 ± 2.35 a	13.33 ± 2.54 a	9.50 ± 3.85 a	1.17 ± 0.17 b

Values are the mean ± SE (*n* = 6). In each row, values followed by the same letter for the different materials (OR, GR) are not significantly different, *p <* 0.05. n.m.: not measured due to insufficient data values.

**Table 4:** Physicochemical properties of the raw olive-mill residues (ORs) and grape-mill residues (GRs).

	OR 100%	GR 100%
pH	6.57	7.19
EC (μS cm^−1^)	1006.60	767.05
Organic matter (%)	95.15	92.70
Organic C (%)	55.19	53.77
C/N ratio	69.00	26.20
N%	0.82	2.05
N (g kg^−1^)	8.19	20.53
P (g kg^−1^)	0.65	2.08
K (g kg^−1^)	6.23	9.86
Ca (g kg^−1^)	6.77	9.75
Mg (g kg^−1^)	0.76	1.24
Na (g kg^−1^)	0.28	0.13
Total porosity (% *v*/*v*)	80.40	99.59
Air-filled porosity (% *v*/*v*)	10.00	33.57
Bulk density (g cm^−3^)	0.34	0.50
Container capacity (% *v*/*v*)	70.40	66.02

## Data Availability

The original contributions presented in this study are included in the article. Further inquiries can be directed to the corresponding author.
